# The Scope of Pasteur’s Work

**Published:** 1898-08

**Authors:** 


### THE SCOPE OF PASTEUR'S WORK.

A statue has been erected at Melun to the memory of Pasteur by the conjoint efforts of the agriculturists and the veterinary surgeons of the Seine and Marne departments. M. Nocard, director of the veterinary school at Alfort, in an able speech, gave a summary of Pasteur's work. He recalled an expression of an admirer of Pasteur, who declared he could not answer if he were asked which was Pasteur's greatest discovery. M. Nocard believes that no one now would be in the same difficulty. Notwithstanding the immense value of Pasteur's work on fermentations, the silkworm malady, beer and wine ferments, and the important researches on spontaneous generation, etc., no previous work can be compared with the progress resulting from the application of Pasteur's methods to medicine. In consequence of this, etiology, sanitation, and public hygiene have been entirely reconstructed. Hospital gangrene, septicæmia, purulent infection, puerperal fever have been banished from hospital wards. Thanks to Pasteur's teaching, surgery is now so safe that the most daring operations can be performed with little risk. Then there is the prophylaxis of infectious diseases founded on the attenuation of viruses. M. Nocard believes that, notwithstanding all that has been achieved, we are only at the beginning of an era which will render Pasteur's name still more glorious. The veterinary profession and the agriculturists were the first who benefited by Pasteur's work. The periodic and repeated losses among livestock have been checked by preventive vaccination, and the past disasters are scarcely remembered. The new and brilliant discovery of serum-therapy is of service in veterinary medicine. It is the best treatment for certain equine affections, especially tetanus, whether surgical or otherwise.—*Bristol* (England) *Medical News.*

